# Biogeomorphic Feedbacks Triggered by Mangrove Degradation at the Seaward Margin Accelerate Persistent Vegetation Decline

**DOI:** 10.1002/ece3.73574

**Published:** 2026-04-30

**Authors:** Longlong Du, Yijuan Deng, Lin Zhang, Zifeng Luo, Mao Wang, Wenqing Wang

**Affiliations:** ^1^ Key Laboratory of the Ministry of Education for Coastal and Wetland Ecosystems Xiamen University Xiamen Fujian China; ^2^ National Observation and Research Station for the Taiwan Strait Marine Ecosystem Xiamen University Zhangzhou Fujian China

**Keywords:** extreme disturbance, mangrove degradation, mangrove seaward margin, surface elevation change, vegetation–geomorphology feedback

## Abstract

Mangrove ecosystems are vulnerable to extreme events and sea‐level rise. The present study examined how biological and geomorphological processes interact at mangrove seaward margins (MSM) using remote sensing, field surveys, and elevation monitoring from 2009 to 2021 in northeastern Hainan, China. The results showed that the mangrove edge had retreated by 11.04 ± 0.36 m. Retreat was more rapid in heavily disturbed areas, especially during 2013–2017 when intense tropical cyclones occurred. In these areas, edge seedling recruitment of *Rhizophora stylosa* declined, with establishment probability being strongly influenced by surface elevation. Mature trees exhibited increased root damage and reduced leaf chlorophyll content, with trait variation primarily driven by surface elevation and sediment physical properties. Following vegetation dieback, surface elevation declined rapidly, forming a positive biogeomorphic feedback that further inhibited regeneration and accelerated margin degradation. We identified a feedback loop between vegetation loss and geomorphic change triggered by extreme disturbances at the MSM that limits natural recovery and threatens ecosystem stability. Our findings underscore the need to prioritize monitoring edge zones and suggest a management framework that integrates remote sensing with in situ monitoring to identify vulnerable zones and guide post‐disturbance conservation and restoration under increasing environmental stress.

## Graphical abstract

Seaward mangrove edge exhibited significant retreat after extreme disturbances. *Rhizophora stylosa* exhibited impaired regeneration and root damage in heavily disturbed areas, trait variation primarily responded to elevation and sediment properties, vegetation loss triggered subsidence, creating a positive biogeomorphic feedback loop. Biogeomorphic feedbacks impaired mangrove recovery and stability.

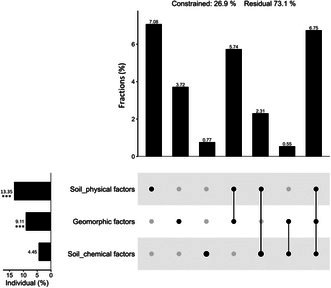

## 1 Introduction

Mangroves are unique coastal ecosystems occurring in tropical and subtropical regions, with a distribution spanning the coastlines of over 100 countries worldwide (Duke et al. 2007; Alongi 2009; Tomlinson 2016). Located at the transition zone between land and sea, mangrove plants have evolved unique adaptive features such as vivipary and diverse aerial roots (Alongi 2009; Chen et al. 2023). Mangrove ecosystems form structurally complex wetlands that integrate vegetation, tidal flats, and tidal creeks (Alongi 2008), thereby sustaining biodiversity while stabilizing shorelines and storing blue carbon. Their extensive aboveground root systems provide shelter for crabs, mollusks, and other species and serve as nurseries, feeding grounds, and refuges for fish, thus contributing significantly to biodiversity (Manson et al. 2005; Spalding et al. 2010; Brander et al. 2012). Simultaneously, dense root networks dissipate wave energy, stabilize shorelines through sediment trapping, and reduce coastal erosion (Guannel et al. 2016). Moreover, mangrove ecosystems are important carbon sinks, with the potential to mitigate climate warming caused by excessive greenhouse gas emissions (Mcleod et al. 2011; Duarte et al. 2013). The MSM—a dynamic ecotone between vegetated mangroves and bare mudflats—faces direct open‐sea forces, maintaining equilibrium under wave‐tidal regimes but is prone to rapid species shifts due to frequent disturbances (Duke and Khan 1999; Jia et al. 2014). This critical interface exhibits steep environmental gradients at fine spatial scales, serving as the ecosystem's most climate‐sensitive zone and a pivotal land‐sea transition zone (Gilman et al. 2008).

Mangrove ecosystems are under dual pressures from human activities and global climate change, leading to rapid degradation worldwide (Bryan‐Brown et al. 2020; Das et al. 2025). Land loss due to aquaculture pond construction, agricultural demands, rapid urban expansion, and overexploitation has caused a sharp decline in mangrove areas (Duke 2016). Although the global rate of mangrove area loss has slowed since 2000, land‐use conversion remains a major driver of mangrove decline in many regions, while climate‐related stressors are becoming increasingly important (Goldberg et al. 2020). These climate‐related stressors threaten mangrove persistence through accelerated sea‐level rise, increasing wave energy, and altered precipitation regimes (Gilman et al. 2008; Reguero et al. 2019; Mondal et al. 2026). Numerous studies have indicated that the accelerating rate of sea‐level rise may lead to the damage and degradation of coastal ecosystems such as mangroves (Saintilan et al. 2020; Buffington et al. 2024; Mondal et al. 2024). According to estimates, the mangrove wetland area will decrease by ~46 km^2^ between 2022 and 2050 under the 1.5 and 1 m SLR scenarios (Mondal et al. 2025). In China, despite reports that mangroves will not experience area loss due to sea‐level rise in the coming decades (Fu et al. 2019), 85% of the coastline has been converted to seawalls, roads, and human settlements (Ma et al. 2014; Wang et al. 2020). As a result, there is little room remaining for mangroves to migrate inland, making the seaward margin increasingly sensitive to various environmental stresses. The growth status of mangrove plants has direct and substantial effects on mangrove degradation, which is first reflected in the condition of the plants themselves. When the growth environment of mangrove plants changes or is disturbed, their physiological functions and morphological structures also change accordingly. In recent years, the definition of ecosystem degradation has expanded beyond the plants themselves to include the regeneration capacity of plant communities as an important indicator of degradation (Ghazoul et al. 2015). The loss of ecosystem services and functions of mangroves, including carbon sequestration and biodiversity maintenance, is also an important manifestation of degradation. Currently, the proportion of rare and endangered mangrove plant species is increasing in China and worldwide (Polidoro et al. 2010). Moreover, biodiversity is declining across multiple biological groups in mangrove ecosystems, from specialized invertebrate fauna at the global scale (Cannicci et al. 2021) to local losses of benthic communities in degraded forests (Carugati et al. 2018). The cumulative effects of multiple stresses and the loss of ecosystem functions ultimately lead to a decline in ecosystem resistance to external stresses. Under high‐intensity disturbances, the probability of ecosystem collapse increases, causing irreversible losses.

Biogeomorphic systems are characterized by complex interactions between vegetation processes and geomorphological units across different spatial and temporal scales (Corenblit and Steiger 2009). Mangroves are typical biogeomorphic systems, where the establishment of mangrove vegetation is accompanied by changes in the geomorphological structure of tidal flats (Rivera‐Monroy et al. 2019). Mangrove plants can regulate surface elevation within the system through biological and physical means in conjunction with hydrological dynamics (Krauss et al. 2014), thereby mitigating the risk of sea‐level rise. On one hand, the underground root systems of mangrove plants can anchor the soil and cause it to expand and rise through physical actions (McKee et al. 2007; Sasser et al. 2018). On the other hand, the extensive aboveground root systems of mangrove plants can efficiently trap sediment during tidal activities, and together with the large amounts of plant litter produced, form physical barriers that slow down water flow, reduce sediment resuspension, and accelerate sediment deposition, promoting surface sedimentation (Chen et al. 2018). Changes in mangrove vegetation, such as reduced vegetation density, can significantly affect surface elevation changes. Over time, the dynamic changes in vegetation lead to complex terrain fluctuations within the forest area, which are significantly different from the flat terrain of mudflats (Fu et al. 2019). Vegetation processes in mangroves at different scales are closely related to geomorphological patterns. Lower surface elevation increases the duration of plant submergence, alters the allocation of mangrove biomass, and leads to morphological variations (He et al. 2007). Additionally, phytotoxic compounds such as sulfides accumulate in the sediment due to prolonged oxygen depletion in the water, exerting toxic effects on mangrove plants and affecting their stomatal opening and carbon assimilation rates (Youssef and Saenger 1998). It is precisely because of the complex feedback mechanisms between plants and geomorphology that when a process within the ecosystem is severely disturbed, such as large‐scale vegetation death or sudden changes in sediment dynamics caused by storm surges, the original feedback mechanisms may no longer be sustainable, leading to a shift to other states (Swales et al. 2019; Wimmler et al. 2021).

MSMs represent dynamic land–sea interfaces where biotic processes and geomorphic changes are tightly coupled and where the impacts of extreme events are often first expressed. However, how disturbance‐driven vegetation degradation interacts with elevation dynamics to reinforce long‐term decline at the MSM remains poorly quantified, particularly through field evidence linking vegetation performance to geomorphic processes across spatial and temporal scales. Here, we aim to (1) quantify MSM retreat and post‐disturbance changes in vegetation condition and recruitment, (2) characterize concurrent changes in surface elevation dynamics, and (3) evaluate whether biogeomorphic feedbacks between vegetation loss and elevation change help explain persistent decline at the seaward margin. By integrating multi‐scale vegetation metrics with elevation processes, our study provides a mechanistic basis for early warning monitoring and offers practical guidance for the conservation of vulnerable MSM amid increasing climate extremes.

## 2 Materials and Methods

### 2.1 Study Area

**FIGURE 1:**
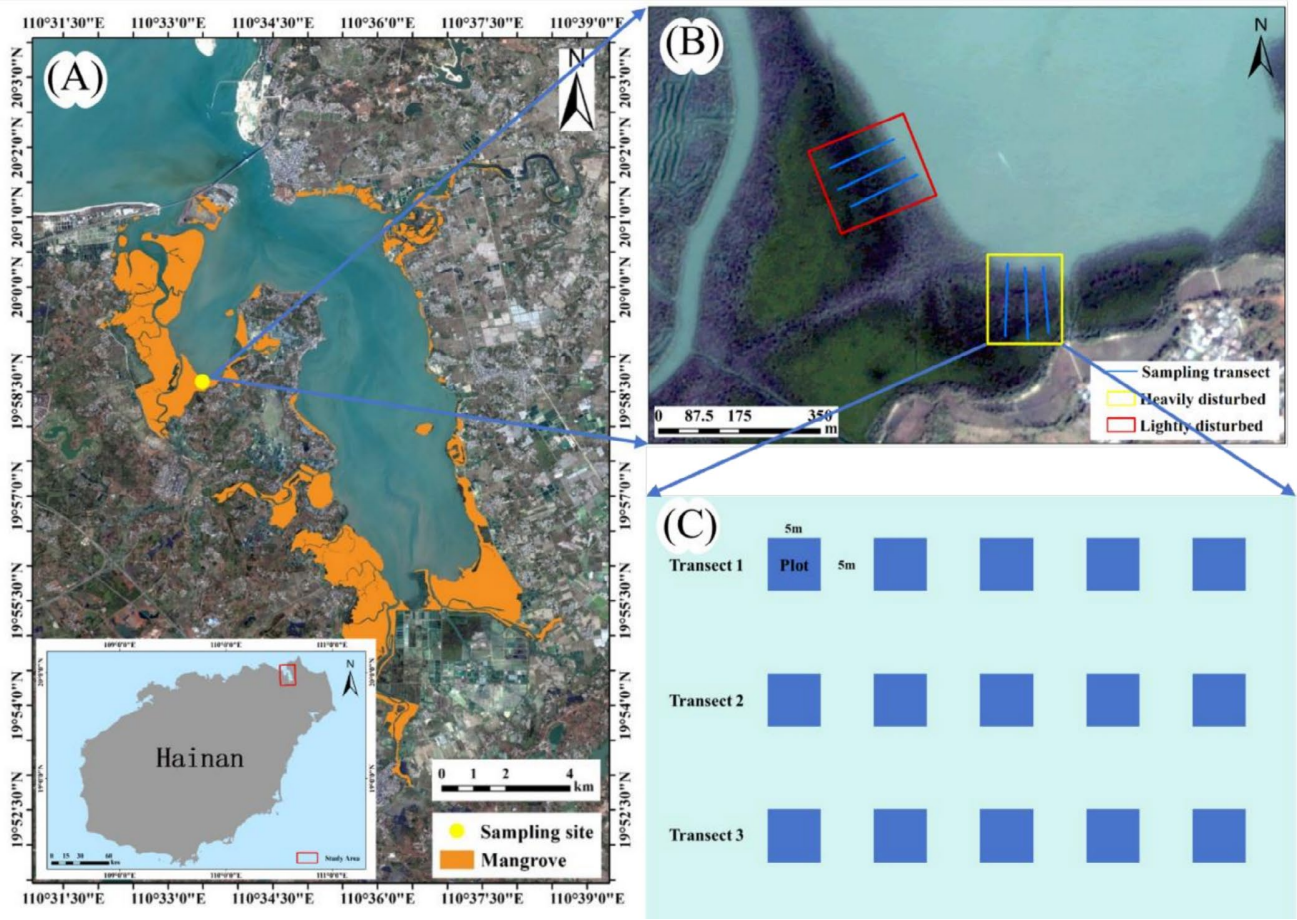
Location of the study area. (A) Location of Dongzhaigang Bay on Hainan Island, China, and the study area within the bay (colored dots); (B) Zonation of the study site into two disturbance levels: Heavily disturbed (HD) and lightly disturbed (LD), based on forest‐edge retreat dynamics and *Avicennia marina* fringe integrity from 2009 to 2021; (C) Sampling transects were arranged perpendicular to the coastline, spanning the full intertidal gradient from seaward to landward margins (100–150 m). Permanent 5 × 5 m quadrats were systematically positioned at 5‐m intervals for vegetation and biogeomorphic monitoring.

This study was conducted in the Dongzhaigang Bay (19°51′–20°01′ N, 110°32′–110°37′ E) on the north‐eastern portion of Hainan Island, China (Figure 1A). This bay is a semi‐enclosed, muddy‐bottom lagoonal bay that receives flow from four rivers and experiences irregular semidiurnal tides with a mean amplitude of 1.6–1.8 m (Fu et al. 2019; Chen et al. 2023). It is the first mangrove reserve established in China and hosts rich mangrove species that show clear zonation from seaward to landward. Historically, the reserve has endured severe disturbances, notably the 2006 *Sphaeroma* infestation (Wang et al. 2017) and the 2014 Cyclone Rammasun (Zheng et al. 2014). These consecutive disturbances have led to marked mangrove degradation in parts of the reserve, with some areas still showing lasting impacts. Within the reserve, the Houpai village area in Yanfeng Town lies in the central part of the bay. Mangroves here exhibit distinct zonation: the seaward fringe is dominated by *Avicennia marina* (AM), followed by a zone of *Rhizophora stylosa* (RS), and a more landward zone of *Ceriops tagal* (CT). Field surveys in 2021 revealed numerous AM stumps with clear signs of insect boring along the seaward edge (Figure S1). In some localized sections, the AM fringe has completely disappeared, exposing the underlying RS stands, while adjacent sections retain a continuous fringe of AM. This spatial heterogeneity makes Houpai an ideal natural experimental site for investigating the effects of extreme disturbances on biogeomorphic processes at the mangrove seaward margin.

### 2.2 Sampling Design

#### 2.2.1 Forest Edge Dynamic Monitoring

This study employed visual interpretation of high‐spatial‐resolution imagery provided by Google Earth Pro to extract the seaward boundary of the mangroves. In Google Earth, cloud‐free and clear images were selected at 4‐year intervals starting from 2009, resulting in the use of four scenes from 2009, 2013, 2017, and 2021. At a sub‐meter spatial resolution, the seaward mangrove boundary was visually interpreted by delineating the interface between vegetation and mudflat (Figure S2), thereby generating a shoreline dataset spanning from 2009 to 2021.

#### 2.2.2 Definition of Disturbance Zones and Transect Establishment

Based on preliminary screening of Google Earth imagery, areas exhibiting severe shoreline retreat between 2009 and 2021, complete mortality of the outer AM fringe, and exposure of inner RS stands to the seaward edge were defined as heavily disturbed areas (HD). Areas with minor shoreline retreat and a persistent outer AM fringe were defined as lightly disturbed areas (LD) (Figure 1B).

The two zones are in close proximity within the same reserve and thus share comparable regional forcing; however, disturbance severity differed markedly between the zones and was associated with *Sphaeroma* (sp.) infestation and the compounding effects of Typhoon Rammasun in 2014.

##### 2.2.2.1 General Characteristics of the HD Zone

The HD zone experienced severe Sphaeroma damage, likely exacerbated by higher sediment salinity and lower elevation, which increased mangrove vulnerability. Shoreline retreat was pronounced during 2009–2021, accompanied by near‐complete loss of the AM fringe and increased exposure of RS at the MSM.

##### 2.2.2.2 General Characteristics of the LD Zone

In contrast, the LD zone exhibited minimal shoreline change and maintained an intact AM front throughout the study period. Although the LD zone was subjected to the same regional stressors, the persistence of the AM fringe functioned as a biophysical buffer, attenuating wave energy and protecting the interior RS. Consequently, the disturbance level here represents a “baseline” of natural resilience compared to the HD zone.

In each zone, three parallel transects (six transects in total) were established perpendicular to the coastline. Each transect extended from the seaward edge landward, with permanent 5 m × 5 m quadrats placed at 5‐m intervals (Figure 1C), resulting in a total of 46 fixed quadrats.

### 2.3 Data Collection and Analysis

#### 2.3.1 Mangrove Forest Edge Extraction and Dynamics

The MSM position was defined as the visible boundary between continuous mangrove vegetation and adjacent unvegetated mudflats and was used as the shoreline proxy for DSAS analyses. Edge lines were digitized manually following a standardized protocol (consistent viewing scale of ~1:1000–1:2000; vertices placed along the canopy edge; single interpreter across years) to minimize operator bias, with digitizing performed at a fine spatial scale (sub‐meter vertex spacing where image quality allowed) to generate an edge‐line dataset for 2009–2021 (Figure S2). Because Google Earth Pro provides static snapshots without concurrent tidal‐stage information, shoreline positions may be influenced by tidal variability, seasonal canopy visibility, georeferencing, and acquisition geometry; therefore, only images with clear canopy–mudflat contrast were used and the proxy definition was kept consistent across years. While residual positional uncertainty may increase the variance in retreat‐rate estimates, the standardized delineation supports robust cross‐year and cross‐zone comparisons.

Shoreline dynamics were quantified using the Digital Shoreline Analysis System (DSASv5.0; US Geological Survey) (Tran Thi et al. 2014), an ArcGIS (Esri, Redlands, CA, USA) extension that automates the generation of shore‐normal transects relative to a user‐defined baseline and calculates rates of shoreline change at transect‐shoreline intersections (Thieler et al. 2009; Himmelstoss et al. 2018). To achieve high spatial resolution, transects were spaced at 2‐m intervals along the baseline, yielding 362 transects. Retreat rates were summarized using the endpoint rate (EPR). EPR was calculated by measuring the distance between the first and last shorelines along each transect and dividing by the time interval between them (Tran Thi et al. 2014). Specifically, let *i* and *j* denote the intersections of a transect with the earliest and latest shorelines, respectively. The baseline‐referenced distances *d*_*i*_ and *d*_*j*_ represent the positional offsets of these intersections. The temporal span *ΔT*_*i,j*_ is defined as the interannual interval (in years) between shorelines *i* and *j*. The shoreline change rate was computed as follows:

(1) $${\mathrm{EPR}}_{\left(i,j\right)}=\frac{d_{j}-d_{i}}{{\mathrm{\Delta T}}_{\left(i,j\right)}}$$

This method provided a quantitative measure of MSM retreat across the study period.

#### 2.3.2 Vegetation Survey and Trait Measurements

A total of 46 quadrats (5 × 5 m) were established across both disturbance zones: 24 in HD and 22 in LD, based on a stratified random sampling design to cover representative microhabitats. Within each quadrat, RS was designated as the focal species for individual‐level growth and trait assessment. Three mature trees per plot were randomly chosen. Real‐Time Kinematic GPS (GPS‐RTK, iRTK10, HiTarget, Guangzhou, China) was used to record their spatial positions and relative ground surface elevations. Morphological traits were quantified as follows: (1) horizontal and vertical root extension ranges; (2) proportion of fractured roots; (3) leaf thickness; (4) leaf area; and (5) foliar water content. Physiological traits measured were the following: (1) SPAD value (chlorophyll content); (2) total organic carbon content; (3) total nitrogen content; and (4) total phosphorus content. Healthy and mature leaves were collected from the upper canopy and analyzed using a scanner (CanoScan LiDE 400, Tokyo, Japan) and an elemental analyzer (Vario EL Cube, Langenselbold, Germany). Horizontal and vertical root extension ranges were measured by excavating roots within a 0.5 × 0.5 m sub‐quadrat and using digital calipers. The proportion of broken roots was calculated as the ratio of visibly damaged roots to total roots. Root density (ind m^−2^) was determined by counting root intersections in a grid. Leaf thickness and foliar water content were measured on five leaves per tree using a micrometer and the oven‐drying method, respectively. SPAD values (chlorophyll content) were obtained with a Konica Minolta SPAD‐502 chlorophyll meter, averaging three readings per leaf from 10 leaves per quadrat. Canopy openness was quantified using hemispherical photography analyzed with Gap Light Analyzer software.

#### 2.3.3 Seedling Recruitment and Survival Monitoring

All RS seedlings within each quadrat were tagged using same‐colored labels, and their survival was subsequently monitored starting in March 2023 (Figure S3). Survival was assessed during follow‐up surveys at 3 and 6 months, and survival rates were calculated for each quadrat at both time points to evaluate early establishment success under local environmental conditions. Because many quadrats contained no seedlings, survival metrics were summarized at the zone level (HD vs. LD) for subsequent analyses. The environmental covariates surface elevation, porewater salinity, and soil properties were measured to assess their effects on seedling survival and establishment success.

#### 2.3.4 Soil and Environmental Factor Collection and Determination

Three replicate surface sediment samples were collected using a soil corer along an S‐shaped path. After on‐site fresh weight measurement, samples were oven‐dried at 60°C to constant weight for bulk density determination. Dried sediments were ground and sequentially sieved through 20‐mesh and 100‐mesh sieves. The < 20‐mesh fraction was analyzed for particle size using a Mastersizer 3000 laser particle size analyzer (Malvern Panalytical Ltd., Malvern, UK) and for pH and salinity using portable devices. The fraction passing the 100‐mesh sieve was analyzed for total organic carbon (TOC) and total nitrogen (TN) using an elemental analyzer (Vario EL Cube) and for total phosphorus (TP) using persulfate digestion and spectrophotometry. Porewater salinity was determined by excavating three randomly distributed mini‐pits (10–20‐cm depth). After allowing porewater to accumulate, three measurements per mini‐pit were taken with a portable salinity meter (Pal‐Salt Probe, Atago, Tokyo, Japan), and the mean value was recorded.

#### 2.3.5 Long‐Term Geomorphological Monitoring and Analysis

We monitored surface elevation change (SEC) and vertical accretion (VA) using rod surface elevation table–marker horizon (rSET‐MH) methodology (Cahoon et al. 2002). Three rSET‐MH benchmarks were deployed in November 2014 across three habitats (AM, RS, CT) within the heavily disturbed area to measure SEC. Each rSET plot contained three 50 × 50 cm MH subquadrats established with powdered feldspar potting clay, enabling concurrent measurement of VA or erosion during the measurement of SEC. The SEC and VA were monitored at 7–12‐month intervals from May 2015 to March 2023 (9‐year period). Shallow subsidence (SS), representing subsurface processes affecting sediment elevation, was calculated as SS = SEC—VA (Cahoon et al. 1995). Surface elevation profiles were acquired using GPS‐RTK surveys, with the seaward edge tree serving as the zero distance point for each transect. Sampling points extended 100 m landward and 100 m seaward at 2‐m intervals, with relative elevation recorded at each point. Integrating rSET plot‐scale measurements with GPS‐RTK surveys enabled comprehensive quantification of elevation dynamics across entire shoreline cross‐sections.

#### 2.3.6 Statistical Analyses

All statistical analyses were performed using R software (version 4.0.2). The significance level for all tests was set at *p* < 0.05. The normality and homogeneity of variance of the data were tested using the Kolmogorov–Smirnov test and Bartlett's test, respectively. Based on these distributions, differences in geomorphic rates (SEC, VA, SS) and vegetation metrics between zones were evaluated using parametric one‐way ANOVA with Tukey's post hoc test or the non‐parametric Wilcoxon rank‐sum test. To disentangle the drivers of degradation, we used Mantel tests to assess the correlations between functional traits (e.g., seedling survival, leaf SPAD, root damage) and environmental matrices (elevation, salinity, and sediment properties). Additionally, Spearman correlation matrices among environmental variables were computed using the “Hmisc” package, and combined plots were generated using the “linkET” package. To quantify recruitment thresholds, logistic regression was applied to model seedling survival probability (binary dependent variable) as a function of relative elevation (continuous independent variable; *n* = 46), with model fit evaluated using 95% confidence intervals and the coefficient of determination (*R* ^2^). Finally, to characterize horizontal topographic structure, we examined the relationship between relative elevation and cross‐shore distance; the 2021 forest edge was used as the shoreline baseline (0 m), and cross‐shore distances were calculated in ArcGIS.

## 3 Results

### 3.1 Mangrove Margin Retreat and Spatial Heterogeneity

**FIGURE 2:**
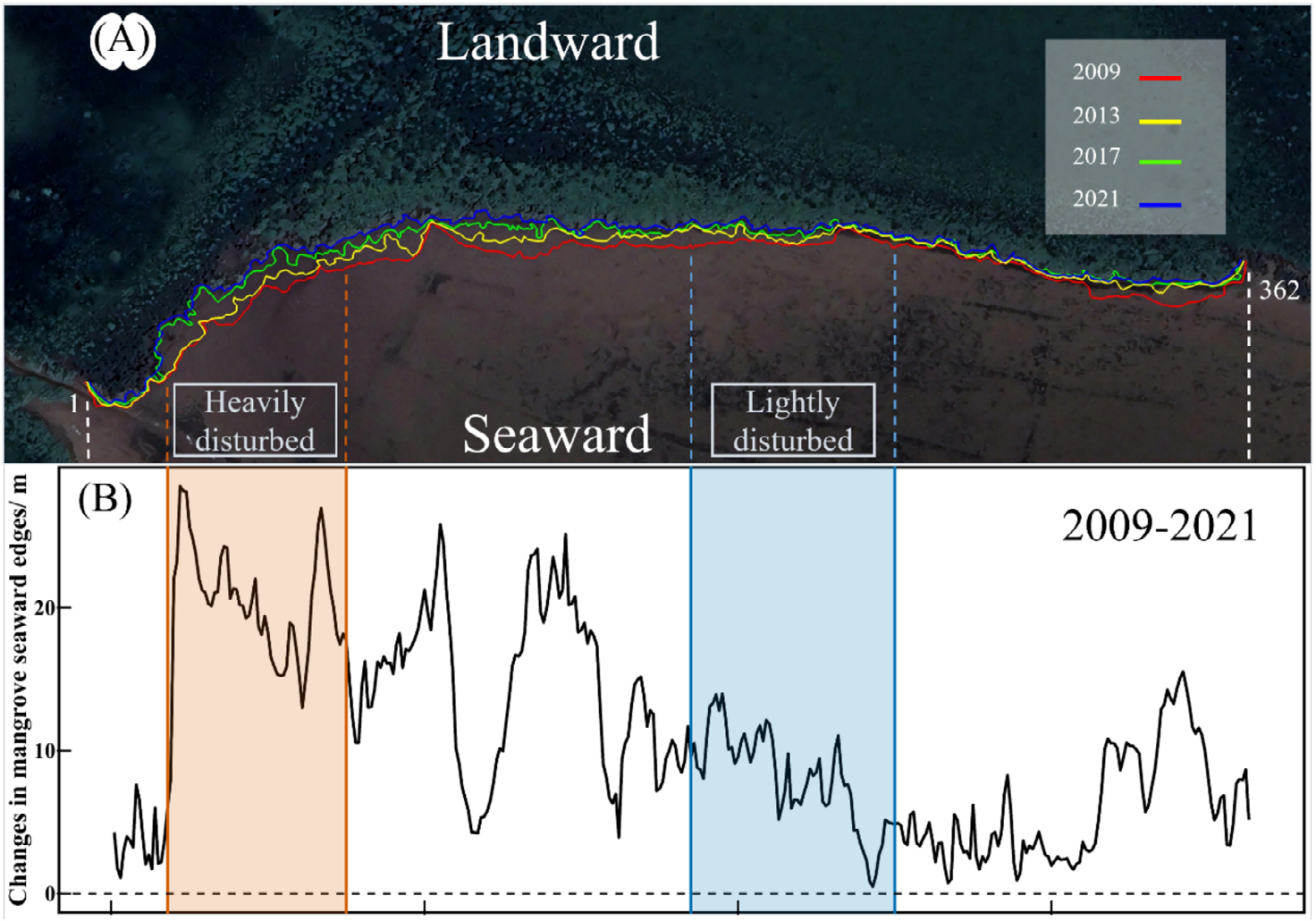
Retreat of the mangrove seaward margin in Dongzhaigang Bay National Nature Reserve, Hainan, China (2009–2021). (A) Interannual dynamics of mangrove forest edges; (B) Average retreat distances in heavily disturbed (HD) and lightly disturbed (LD) areas.

**FIGURE 3:**
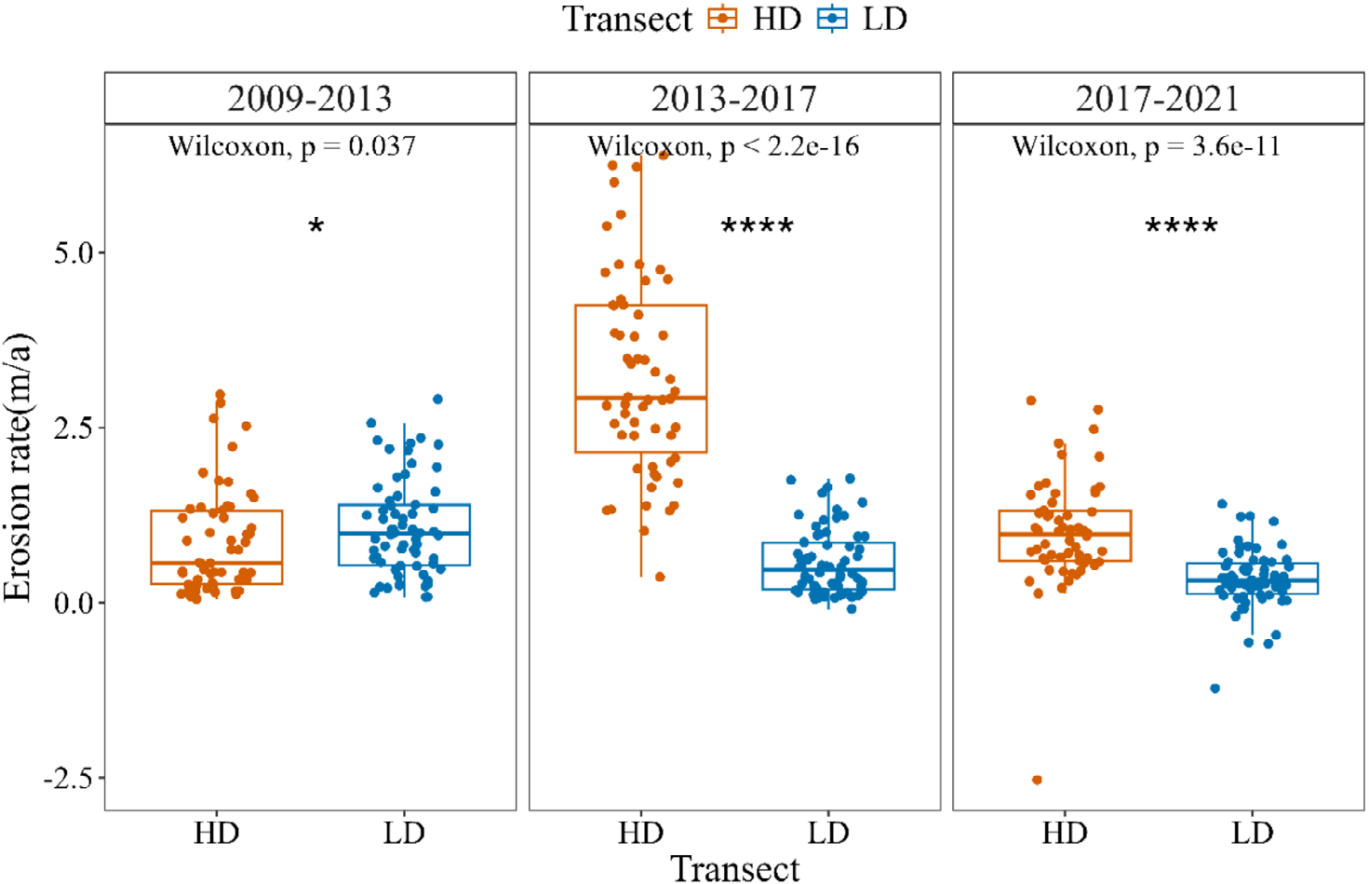
A comparison of the average erosion rates in heavily disturbed areas (HD) and lightly disturbed areas (LD) in different time periods (Annual shoreline retreat rates for HD (Recovery trend observed in HD post‐storm) and LD. Retreat intensified during 2013–2017, coinciding with Cyclone Rammasun).

From 2009 to 2021, the Dongzhaigang (DZG) mangroves exhibited significant seaward margin retreat, with mean shoreline displacement of 11.04 ± 0.36 m (Figure 2A). Retreat magnitudes demonstrated pronounced spatial heterogeneity (range: 0.48–28.49 m), with HD experiencing significantly greater retreat (20.05 ± 0.57 m) than LD (8.04 ± 0.40 m; *p* < 0.01; Figure 2B). Retreat was most pronounced during 2013–2017, with an average rate of 3.23 ± 0.19 m year^−1^ in HD (Figure 3), which is temporally aligned with the 2014 landfall of Cyclone Rammasun. In the post‐storm period, erosion rates in HD decreased to near pre‐storm levels (from 8.2 to 3.5 m year^−1^), suggesting partial recovery, but remained significantly higher than LD (*p* < 0.05), indicating persistent vulnerability. Unless otherwise stated, values are presented as mean ± standard error (SE).

### 3.2 Variation of *Rhizophora stylosa* at the Edges of Different Degradation Zones

**FIGURE 4:**
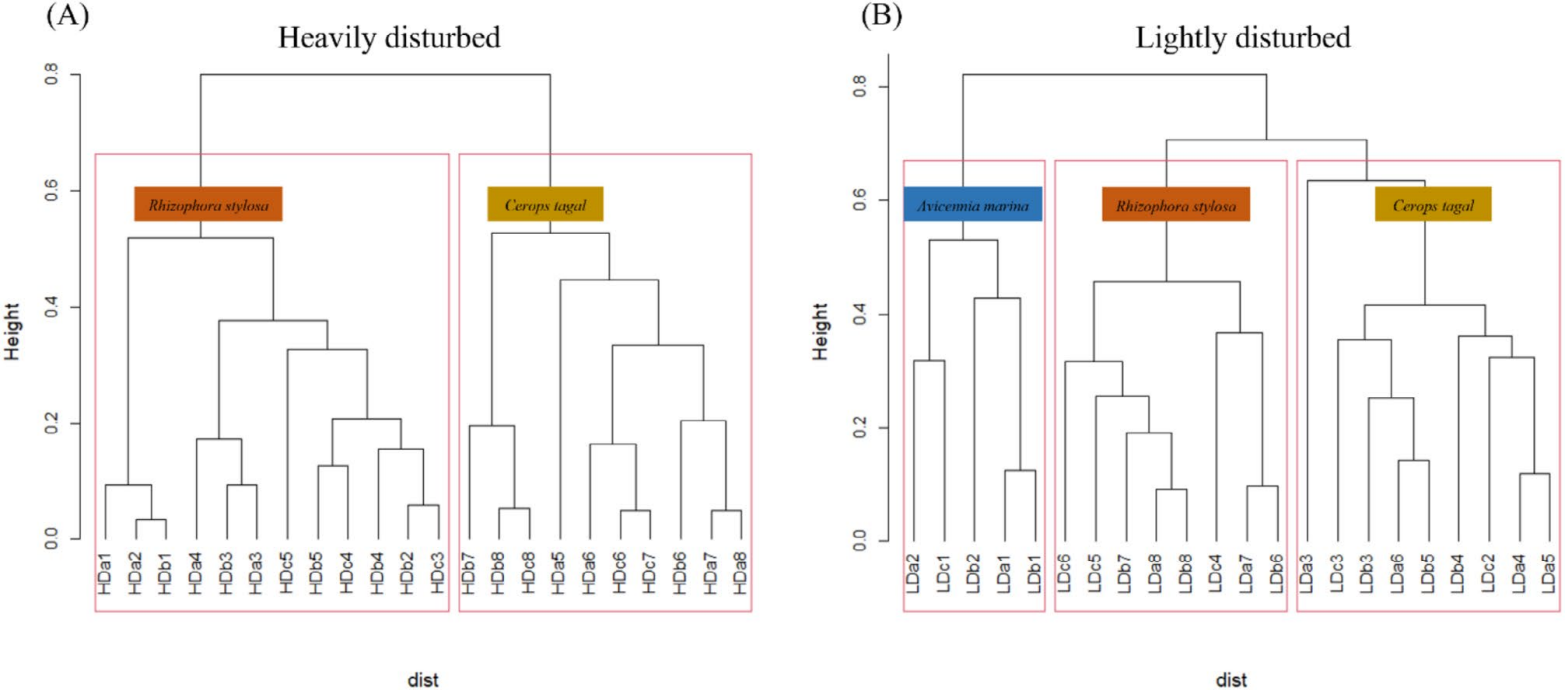
Plant community structure of heavily disturbed area (A) and lightly disturbed area (B). (AM, *Avicennia marina* ; CT, *Ceriops tagal* ; RS: *Rhizophora stylosa*).

The community pattern was simplified in the heavily disturbed zone (Figure 4). The pioneer species AM belt, originally located at the outer edge, completely disappeared and was replaced by RS as the new frontline species. This shift toward the margin reduced the regeneration potential of the RS population: juvenile individuals accounted for only 41.75% of the population, a proportion lower than that of adult individuals (Figure S4).

**FIGURE 5:**
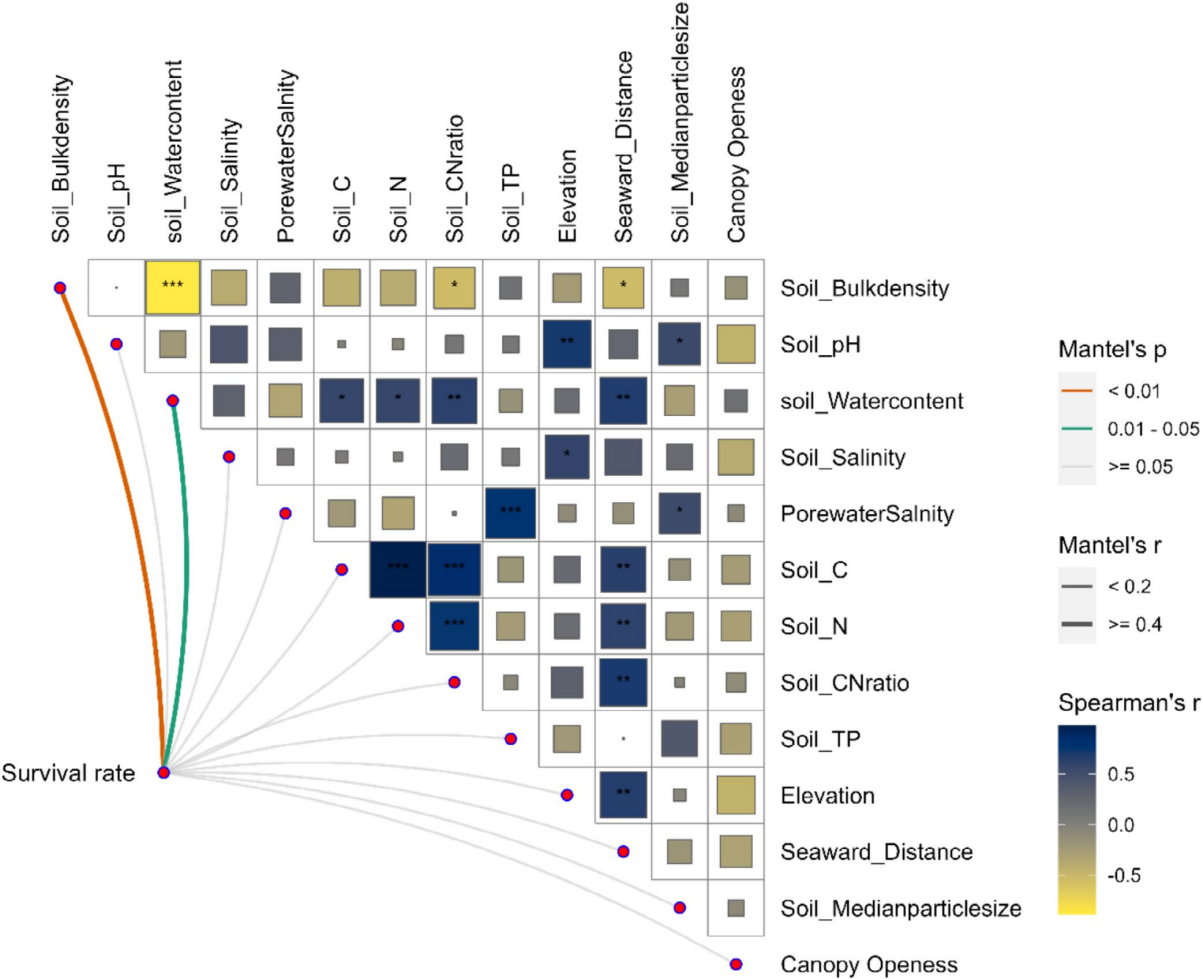
Mantel test of *Rhizophora stylosa* seedling survival rate with environmental factors and Spearman correlation matrix among environmental factors. Curved lines indicate Mantel correlations between seedling survival rate and environmental factors, with line color representing Mantel's *p* value and line width representing Mantel's r. Colored squares indicate Spearman's correlation coefficients among environmental factors. Asterisks indicate the significance levels of correlations among environmental factors: *0.01 < *p* < 0.05; **0.001 < *p* < 0.01; ***p < 0.001.

In the tagged seedling monitoring trial (Figure S5), the 3‐month survival rate of RS seedlings did not differ significantly between zones, averaging 31.0% ± 11.4% (mean ± SD) in HD versus 35.8% ± 17.8% in LD. By 6 months, however, survival in HD declined significantly to 2.6% ± 1.7%, compared with 30.0% ± 16.3% in LD (*p* < 0.05). Mantel analysis revealed significant correlations between seedling survival and sediment bulk density (*r* = 0.41, *p* < 0.01) and moisture content (*r* = 0.48, *p* = 0.02) (Figure 5). The strong correlation between sediment bulk density and moisture content (*p* < 0.01) suggested their joint role as primary drivers of seedling mortality.

### 3.3 Trait Variation in Mature Trees

**FIGURE 6:**
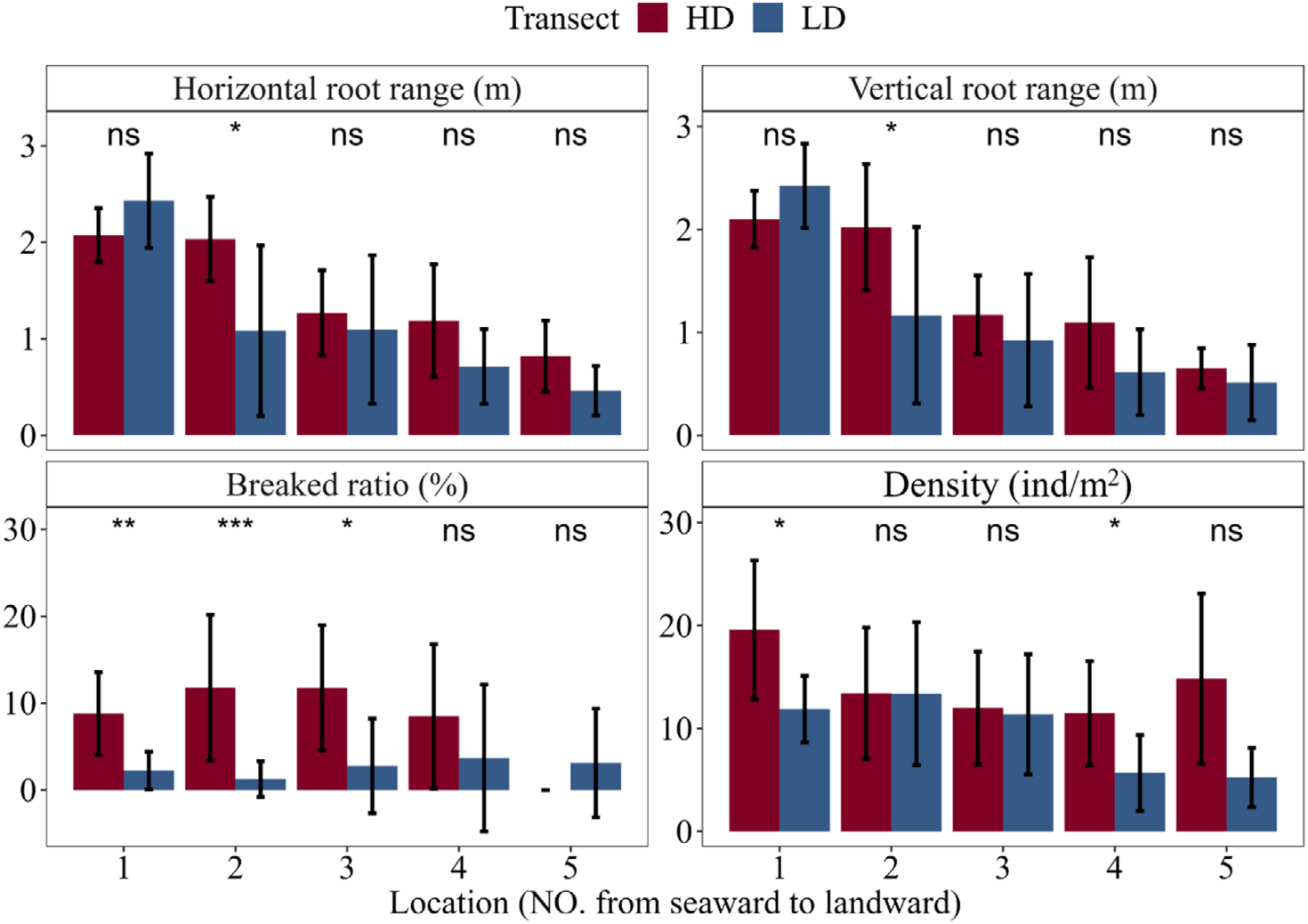
Differences in the root structure of *Rhizophora stylosa* in the heavily disturbed area (HD) and the lightly disturbed area (LD) (Numerical increases indicate seaward to landward). ns, not significant; * *p* < 0.05; ** *p* < 0.01; *** *p* < 0.001.

Root damage incidence in mature RS was significantly higher in HD than in LD across all plots (Figure 6). The two shoreline‐proximal plots showed the most pronounced divergence, with damage rates of 8%–12% in HD versus 2.25% ± 2.17% in LD (*p* < 0.01). Across both zones, the proportion of plants exhibiting root damage was 77.78% in HD, significantly higher than the 43.6% observed in LD (*p* < 0.01). Leaf SPAD values were lower in HD than in LD (Figure S6), with significant differences in the second and third plots (*p* < 0.05).

**FIGURE 7:**
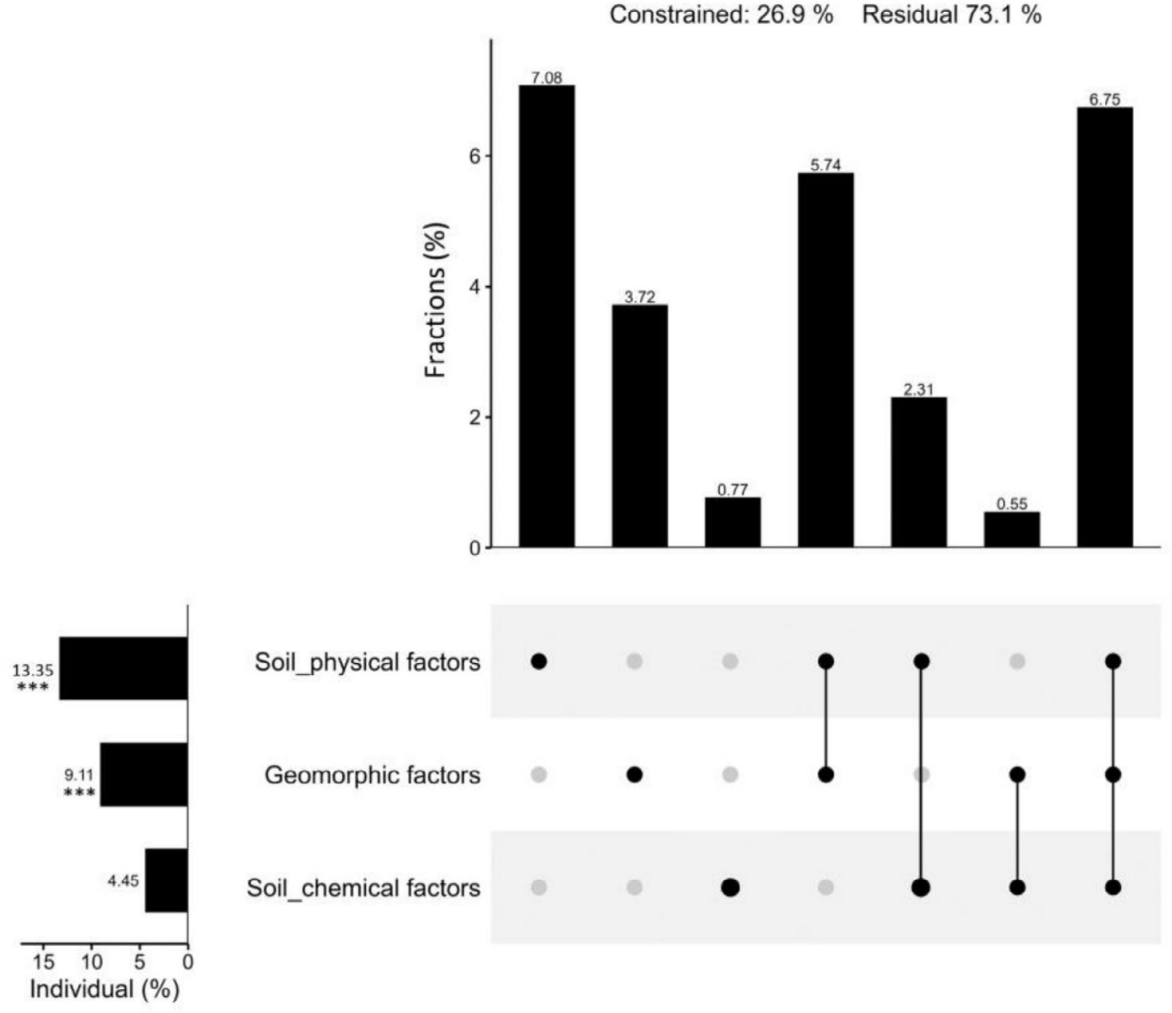
Relative importance of geomorphic, physical, and chemical factors of sediments on trait variation of *Rhizophora stylosa*.

Variation partitioning analysis revealed that geomorphic characteristics (9.11%) and sediment physical properties (13.35%) together explained a significant portion of the observed trait variation, with surface elevation being the most important single factor (Figure 7).

### 3.4 Changes in Biogeomorphic Patterns

#### 3.4.1 Geomorphic Processes and Temporal Variation Across Vegetation Zones

**FIGURE 8:**
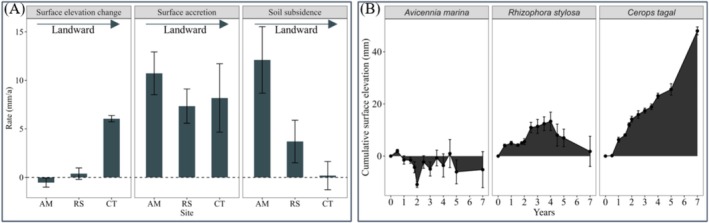
Magnitude of surface elevation change. (A) Rates of surface elevation change, surface accretion, and soil subsidence at different sites in the heavily disturbed zone (AM, *Avicennia marina* ; CT, *Ceriops tagal* ; RS, *Rhizophora stylosa*); (B) Cumulative surface elevation change at different sites (from left to right: AM, RS, CT).

The SEC rates exhibited significant spatial heterogeneity across tidal zones (*p* < 0.01, ANOVA). At the low‐tide zone, the AM site showed an average annual elevation decline of −0.53 ± 0.47 mm year^−1^ (Figure 8A). The mid‐tide RS site had slight accretion of 0.38 ± 0.59 mm year^−1^, while the high‐tide CT site accreted 6.05 ± 0.32 mm year^−1^.

The VA rate was 10.73 ± 2.99 mm year^−1^ at the AM site, slightly higher than that at RS (7.35 ± 1.76 mm year^−1^) and CT (8.18 ± 3.53 mm year^−1^) sites. The SS rates decreased substantially from sea to land: 12.11 ± 3.43 mm year^−1^ at AM, 3.70 ± 2.20 mm year^−1^ at RS, and 0.18 ± 1.46 mm year^−1^ at CT. Over 7 years of monitoring (2015–2022), sustained subsidence with a cumulative elevation loss of 5.19 mm was observed at the AM site (Figure 8B). By contrast, progressive sediment accretion occurred at the CT site, accumulating a total of 48.03 mm. The RS site displayed biphasic elevation dynamics, with an initial gain of 13.34 mm (2015–2019) followed by rapid subsidence of 11.54 mm (2019–2022), resulting in a marginal net increase of 1.80 mm (Figure 8B).

#### 3.4.2 Vegetation–Geomorphology Interaction

**FIGURE 9:**
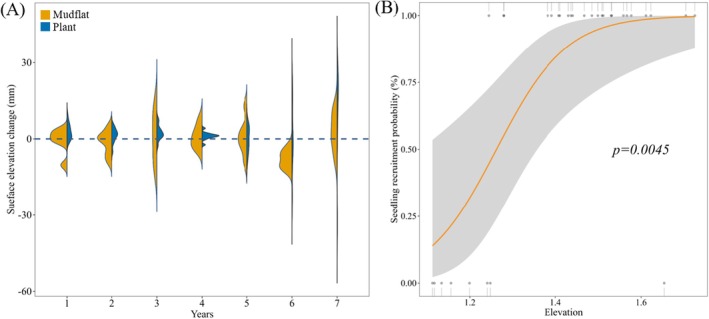
Biogeomorphic feedbacks and elevation thresholds for seedling establishment. (A) Elevation fluctuation in vegetated and adjacent mudflat zones in the highly disturbed area over time. (B) Logistic regression model showing the probability of seedling establishment across different surface elevations. The critical recruitment threshold occurs at 1.2–1.4 m.

In HD, divergent elevation trajectories emerged between vegetated and unvegetated habitats. Forested areas exhibited a consistent accretion trend, driven by root network stabilization and organic matter accumulation. By contrast, bare flats experienced high‐frequency sediment oscillation. This result demonstrated that vegetation‐mediated biophysical feedbacks increased sediment retention and substrate stability. During the initial 4‐year period (2015–2018), the RS forest maintained stable elevation dynamics. However, after 2019, extensive root damage severely compromised sediment retention capacity, triggering a net erosion phase (Figure 9A). Logistic regression analysis revealed a significant positive relation between elevation and seedling establishment probability (*p* = 0.0045). A critical elevation threshold was identified at 1.2–1.4 m, with establishment probability remaining below 25% at elevations ≤ 1.2 m but increasing exponentially to exceed 75% at elevations ≥ 1.4 m (Figure 9B).

## 4 Discussion

### 4.1 Drivers of Tree Mortality

The MSM in the DZG experienced peak degradation during 2013–2017, coinciding with landfall of Cyclone Rammasun. The storm damaged 21.31% of reserve mangroves, causing severe habitat destruction (Zhang et al. 2022; Gao et al. 2025). The magnitude of degradation exhibited significant spatial heterogeneity: HD experienced over 2.5 times as much shoreline retreat as LD (Figure 3), indicating that disturbance intensity modulates mortality patterns. AM suffered greater damage than RS, potentially linked to precyclone *Sphaeroma* infestations from agricultural pollution (Wang et al. 2017). Bio‐eroded AM stumps were extensive in HD, in contrast to no invertebrate damage in LD. Chronic stressors systemically compromised structural integrity, reducing storm resilience and impeding natural recovery (Walcker et al. 2019). Post‐disturbance recovery trajectories vary from months to decades, with some sites showing continued degradation (Aung et al. 2013).

No mangrove recovery occurred at the MSM within the 7 years post‐disturbance. By 2023, RS in HD exhibited functional trait alterations (e.g., reduced SPAD, elevated root damage), particularly near eroding shorelines. This persistent degradation reflects a “memory effect” from cyclone‐induced structural compromise (Krauss et al. 2014), which progressively impairs physiological function (Lovelock et al. 2015) and suppresses sediment accretion below erosion thresholds. The AM zone in LD functioned as a biogeomorphic buffer, attenuating wave energy and shielding interior RS, consistent with global mangrove fringe protection paradigms (Alongi 2008). Resistance varies by species (Imbert 2018) and forest structure (Sherman et al. 2001; Rivera‐Monroy et al. 2019) further explains spatial heterogeneity in storm resilience. For example, AM branches have higher shear strength than those of *Bruguiera gymnorrhiza* (van Hespen et al. 2021), contributing to species‐specific resistance to storms.

Root damage (Mantel tests: *p* < 0.05) was linked to low elevation, proximity to the sea, and elevated sediment salinity, which induced hypoxia and physiological dysfunction. Proximity to tidal creeks—key pathways for pollutant transport and sites of higher *Sphaeroma* densities—intensified biotic damage in HD. Here, widespread root damage (77.78%) and reduced photosynthesis prevented metabolic maintenance, triggering delayed mortality and regeneration failure, as evidenced by catastrophic failure of seedling survival (2.56% at 6 months). RS root damage declined along elevation gradients, consistent with mangrove zonation paradigms (Wang et al. 2020), where elevation governed flooding duration and sediment properties (salinity, bulk density, moisture; all *p* < 0.01). Storm‐induced sediment displacement and salt accumulation elevated physiological stress, root damage amplified rhizosphere degradation through salinization and hypoxia, establishing a self‐reinforcing biogeomorphic feedback. Recovery hinges on the dual processes of survival of established stands and successful seedling recruitment (Krauss and Osland 2020). Chronic constraints (low elevation, high salinity, and tidal inundation) collectively inhibit MSM regeneration and growth. Physiological thresholds are breached during extreme events, triggering irreversible degradation, as evidenced by catastrophic low seedling survival (< 3% over 6 months), preventing natural population renewal and driving local extinction. Spatial heterogeneity in degradation intensity reveals differential community sensitivity, with the seaward margin prone to entering a “critical degradation zone” that poses serious challenges for future recovery.

### 4.2 Vegetation Degradation Alters Geomorphic Processes

In mangrove ecosystems, plants and geomorphology form stable interaction networks: healthy vegetation promotes sediment accumulation and elevation gain through root stabilization, branch sediment trapping, and litter build up, maintaining suitable tidal elevation zones (Krauss et al. 2014). Extreme events trigger systemic MSM mortality, collapsing root structures and inducing rapid sediment compaction—consistent with post‐disturbance geomorphic responses (Cahoon et al. 2003). Crucially, root systems regulate surface elevation via: (1) dense aerial roots decelerate tidal flow to enhance sediment capture (van Maanen et al. 2015); (2) porous underground networks (high root volume fraction), reducing compaction while adding organic matter (Pi et al. 2009; Sasser et al. 2018); (3) root death and altered sediment texture driving surface collapse (Cahoon et al. 2003; Lang'at et al. 2014).

Post‐disturbance geomorphic trajectories showed significant spatial heterogeneity between adjacent HD and LD sites. Over 90 months, elevation dynamics diverged across HD sites: AM and RS exhibited minimal change (< 1 mm year^−1^), with no net gain, whereas CT showed an increase in elevation. This contrasts sharply with mainland Chinese mangroves, where land‐derived sediment and high suspended particle concentrations drive rapid elevation rise (Fu et al. 2019). In other areas of Hainan (Sanjiang and Tashi), elevation increases rapidly and then decreases gradually from sea to land, in contrast to our finding of higher elevation landward and lower seaward.

Generally, increased tidal flooding elevates sediment deposition but intensifies self‐compaction (Gilman et al. 2007; Rogers and Saintilan 2021). Excessive flooding alters sediment texture (Rogers and Saintilan 2021), and without vegetation, subsidence dominates sedimentation gains. Post‐AM mortality, sites transitioned to bare flats with highly dynamic elevation fluctuations driven by rainfall, sediment pulses, and tidal forcing. Crucially, vegetation dampens environmental variability, stabilizing elevation through biophysical feedback.

Dynamic elevation instability in extant RS at seaward margins of HD reflects differential sediment trapping capacities mediated by root morphotypes. Prop roots are superior at sediment accretion compared with those of stilt and knee roots in Micronesia (Cahoon et al. 2003; Du et al. 2021), while plate roots promote sedimentation most in Guangxi mangroves, followed by knee and stilt roots (Du et al. 2021). Notably, stilt roots prevail in prograding coastlines (Vietnam), in contrast to prop roots in eroding systems (Nguyen et al. 2023). Structurally, stilt roots (*Avicennia* and *Sonneratia*) increase flood resilience via high porosity and radial oxygen loss (ROL), sustaining aerobic rhizospheres and nitrogen capture under flooding (Cheng et al. 2015, 2020). This functional adaptation underscores their stress tolerance superiority over prop roots.

Seaward‐to‐landward gradients revealed divergent root dynamics. Root types exhibit distinct zonation patterns along elevation and inundation gradients in the intertidal zone (Chen et al. 2023). Chronic unsuitable conditions damage root structures, progressively compromising sediment‐trapping capacity. Although damaged roots typically regenerate via wound periderm formation (Hendy and Cragg 2017), intensified flooding disrupts the aerobic rhizosphere, lowering nutrient uptake and impairing repair mechanisms, leading to root fragmentation and sediment destabilization. Consequently, seaward–landward elevation disparities amplify intertidal slope steepness over time, inhibiting seedling establishment (van Bijsterveldt et al. 2020).

**FIGURE 10:**
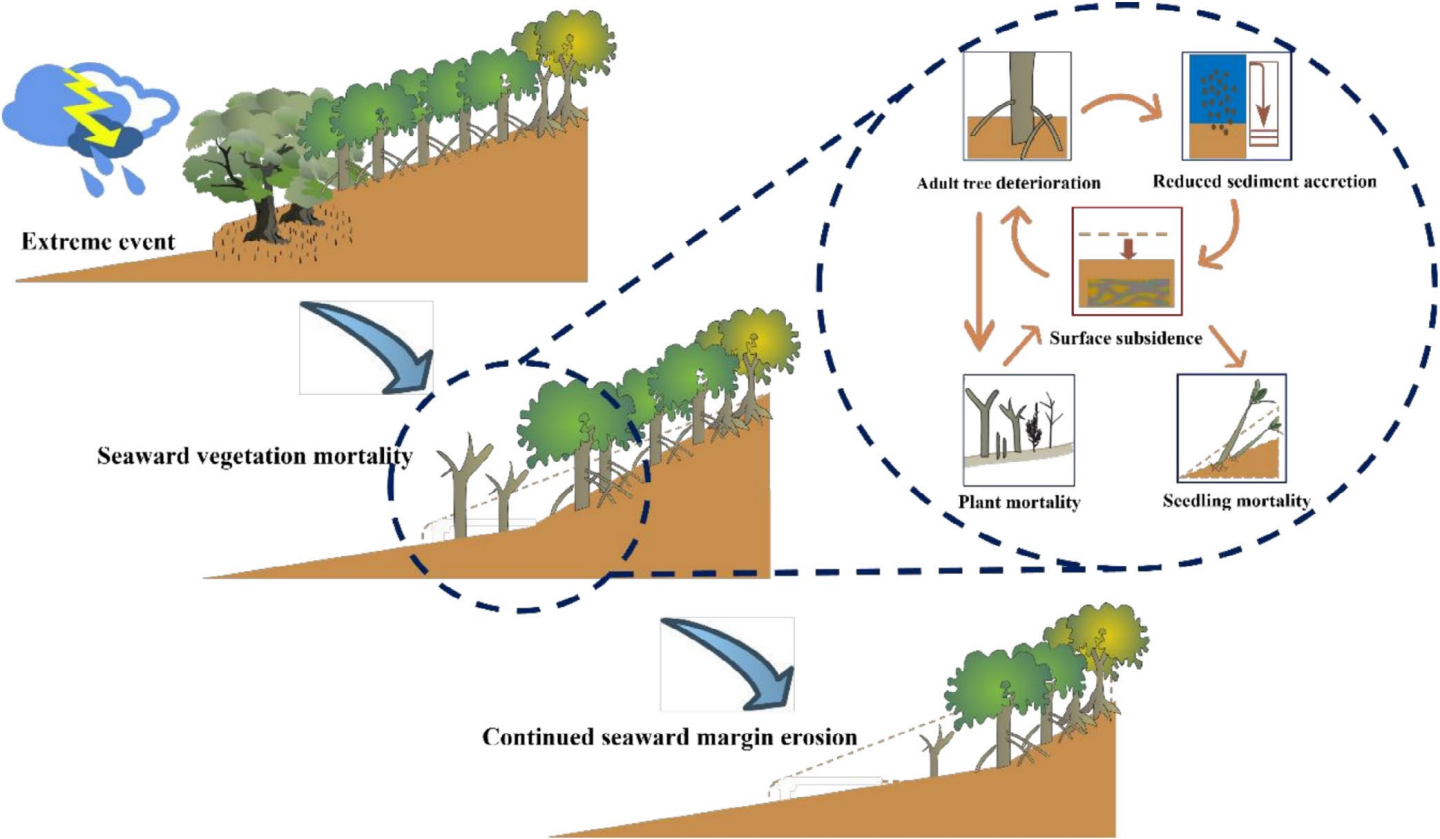
Conceptual model of the response of the mangrove seaward margin to extreme events. Summary: Disturbance triggers vegetation loss, leading to elevation decline and sediment erosion, which inhibit seedling recruitment and perpetuate degradation. Potential outcomes include irreversible collapse if elevation thresholds are breached, or recovery via restoration interventions.

Overall, this study reveals complex biogeomorphic feedbacks between vegetation and geomorphology through tidal processes. Following extreme disturbances, initial mortality among MSM (e.g., AM) triggers rapid degradation, leading to sediment textural shifts and surface collapse. These altered conditions impair RS regeneration and degrade functional traits (e.g., root architecture), further accelerating surface erosion and environmental stress. Once *Rhizophora* function declines beyond tolerance, plants die, causing local surface collapse, and perpetuating this positive feedback loop (Figure 10).

### 4.3 Vulnerability Assessment and Management Implications for Mangrove Edges

The MSM functions as a dynamic ecological interface and is therefore a biogeomorphic risk hotspot. Under landward development and spatial compression, future mangrove persistence hinges on the capacity for seaward expansion, making edge stability a critical determinant of ecosystem fate (Alongi 2009; Friess et al. 2019). The MSM exhibits heightened vulnerability to extreme events and reduced resilience capacity. Remote sensing signatures of degradation (Figure 2A) delineate high‐risk zones characterized by rapid forest edge retreat, severe root structural damage, and sustained elevation loss, necessitating priority ground monitoring and ecological intervention.

However, some early‐stage or subtle degradation indicators may be missed by broad‐scale or cover‐change–based remote sensing products alone, especially in tidally dynamic margins where canopy signals can be obscured or temporally variable. At the same time, we acknowledge that recent advances in remote sensing—such as high‐resolution optical imagery, optical–SAR time series, structural observations, and machine‐learning approaches—increasingly detect many forms of “hidden” or pre‐canopy degradation. Nevertheless, these approaches still benefit from field validation and process‐based measurements, particularly where elevation dynamics and substrate conditions directly constrain regeneration. Accordingly, we propose a two‐tiered assessment strategy: first, use remote sensing to identify exposed, high‐risk zones with clear degradation; second, combine long‐term elevation tracking with leaf traits and seedling establishment surveys in areas where imagery shows no change. This approach can help to identify critical “vulnerability points” before irreversible biogeomorphic transitions, facilitating timely interventions to enhance ecosystem resilience.

We identified multiple interacting drivers of MSM degradation by using integrated forest edge change analysis, adult and seedling surveys, and rSET‐MH elevation analysis. Our results confirm that retreat areas observed by remote sensing accurately reflect geomorphic and ecological instability. We propose an adaptive management framework that prioritizes edge protection through “remote sensing detection–elevation modeling–ecological restoration”. Specifically, edge dynamics should be monitored with high‐resolution remote sensing and DSAS to identify high‐risk retreat zones; rSET‐MH elevation tracking should be integrated to capture “hidden degradation” signals; and crucially, to break the identified vegetation loss–sediment erosion feedback loop, intervention protocols must be threshold‐based. In areas where elevation has exceeded the critical threshold for recruitment, managers must prioritize substrate stabilization (e.g., through sediment nourishment or permeable dams) to restore suitable hydroperiods before attempting reforestation.

We advocate shifting from species‐centric planting to systemic restoration, utilizing a site‐specific approach that considers species selection, habitat matching, and biogeomorphic processes. Our findings highlight the MSM as a particularly sensitive ecosystem segment and show that coupling remote sensing of edge dynamics with rSET‐MH–based elevation tracking offers a scalable template for broader coastal monitoring under sea‐level rise and increasing disturbance pressures. This multi‐metric approach helps address key limitations of optical remote sensing by enabling the early detection of “invisible” degradation signals, supporting a shift from reactive emergency responses to proactive resilience management. Looking ahead, future research should integrate hydrodynamic modeling to quantify physical erosion thresholds and test species‐specific tipping points of inundation tolerance. Addressing these gaps will further refine predictive vulnerability assessments and inform precision restoration strategies worldwide.

## 5 Conclusions

Our study indicates that extreme disturbances at MSM can initiate a rapid, self‐reinforcing biogeomorphic feedback loop that contributes to persistent vegetation decline. By integrating remote sensing with in situ monitoring, we showed that high‐intensity disturbance coincided with faster shoreline retreat and marked deterioration in *Rhizophora stylosa* performance, together with unfavorable elevation/sediment conditions that constrained seedling establishment, consistent with a threshold‐like response. Although site‐specific and largely correlative, our results point to a tipping‐point mechanism in which geomorphic instability outpaces biological recovery. Accordingly, changes in canopy‐cover alone may miss early‐stage degradation; combining edge‐change detection with elevation tracking and functional indicators could improve early warning and post‐event assessment. Future work should couple higher‐frequency remote sensing with hydrodynamic and sediment measurements to test causal pathways, while restoration at eroding margins may need to prioritize substrate stabilization and hydrological remediation to alleviate elevation deficits before reforestation under increasing climate extremes.

## Author Contributions

**Longlong Du:** conceptualization (lead), data curation (lead), formal analysis (lead), investigation (lead), methodology (equal), writing – original draft (equal), writing – review and editing (lead). **Yijuan Deng:** data curation (equal), formal analysis (equal), investigation (equal), methodology (lead), visualization (lead), writing – original draft (lead), writing – review and editing (equal). **Lin Zhang:** data curation (equal), formal analysis (equal), investigation (equal), writing – review and editing (equal). **Zifeng Luo:** investigation (equal), writing – review and editing (equal). **Mao Wang:** conceptualization (equal), methodology (equal), supervision (lead), writing – review and editing (equal). **Wenqing Wang:** conceptualization (equal), funding acquisition (lead), methodology (equal), project administration (lead), supervision (equal), writing – review and editing (equal).

## Funding

This work was supported by National Natural Science Foundation of China, 42176169.

## Conflicts of Interest

The authors declare no conflicts of interest.

## Supporting information


**Figure S1:** Dying *Avicennia marina* in Houpai manrgrove seaward margin (A); Biten trunks (B).
**Figure S2:** Mangrove seaward edge extraction results (A: 2009; B: 2013; C: 2017; D: 2021).
**Figure S3:**. In situ tagging and periodic monitoring of naturally recruited mangrove seedlings.
**Figure S4:** Comparison of regeneration status of *Rhizophora stylosa* population in heavily disturbed area (HD) and lightly disturbed area (LD) from seaward to landward (Numerically increasing, ALL represents the whole population).
**Figure S5:** Comparison of survival rates of *Rhizophora stylosa* seedlings in heavily disturbed area (HD) and lightly disturbed area (LD).
**Figure S6:** Differences in SPAD value of *Rhizophora stylosa* in heavily disturbed area (HD) and lightly disturbed area (LD) (Numerically increasing indicates seaward to landward).

## Data Availability

The raw data in this article are available from the Zenodo repository (https://doi.org/10.5281/zenodo.17291535).
