# Supplementary material for: Biogeomorphic Feedbacks Triggered by Mangrove Degradation at the Seaward Margin Accelerate Persistent Vegetation Decline
Source: Ecol Evol. 2026 Apr 30;16(5):e73574. doi: 10.1002/ece3.73574 (PMC13129671; doi:10.1002/ece3.73574)
Supplement: Supplementary file 1 — Figure S1: Dying Avicennia marina in Houpai manrgrove seaward margin (A); Biten trunks (B). Figure S2: Mangrove seaward edge extraction results (A: 2009; B: 2013; C: 2017; D: 2021). Figure S3:. In situ tagging and periodic monitoring of naturally recruited mangrove seedlings. Figure S4: Comparison of regeneration status of Rhizophora stylosa population in heavily disturbed area (HD) and lightly disturbed area (LD) from seaward to landward (Numerically increasing, ALL represents the whole population). Figure S5: Comparison of survival rates of Rhizophora stylosa seedlings in heavily disturbed area (HD) and lightly disturbed area (LD). Figure S6: Differences in SPAD value of Rhizophora stylosa in heavily disturbed area (HD) and lightly disturbed area (LD) (Numerically increasing indicates seaward to landward). [file ECE3-16-e73574-s001.pdf]

Supplementary material

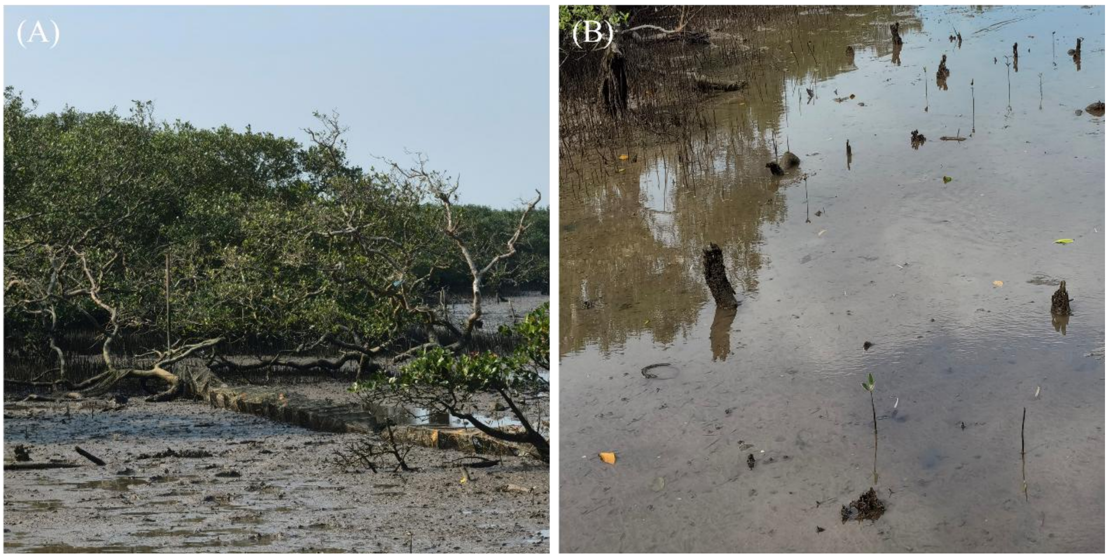

Figure S1 Dying *Avicennia marina* in Houpai manrgrove seaward margin(A); Biten trunks (B)

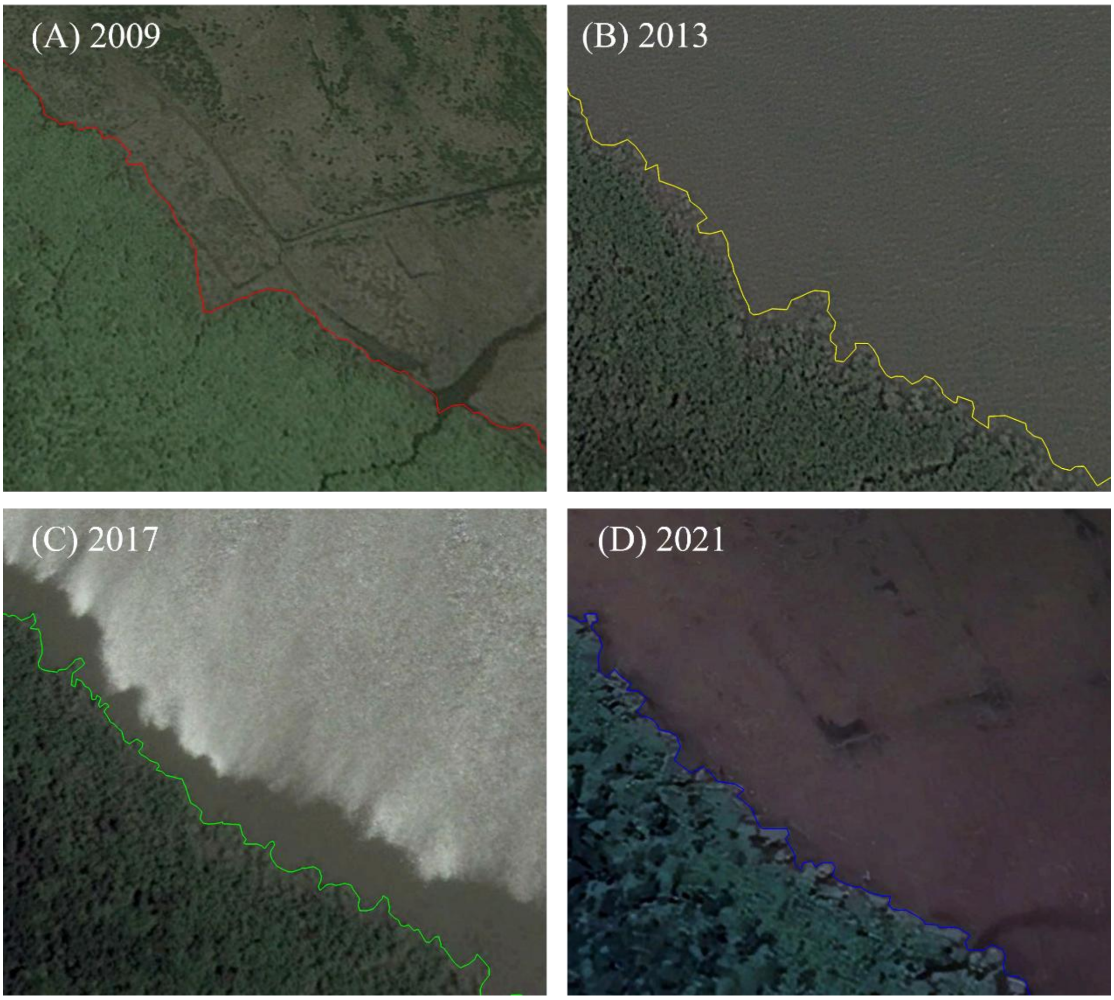

Figure S2 Mangrove seaward edge extraction results (A:2009; B:2013; C:2017; D:2021)

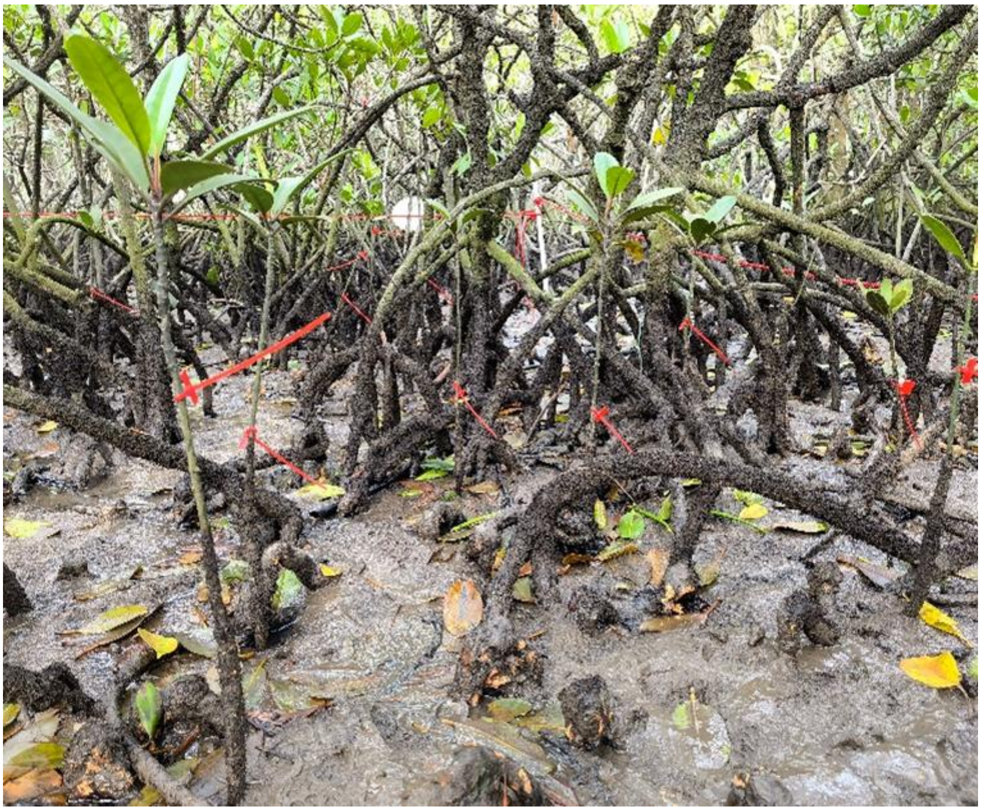

Figure S3 In situ tagging and periodic monitoring of naturally recruited mangrove seedlings

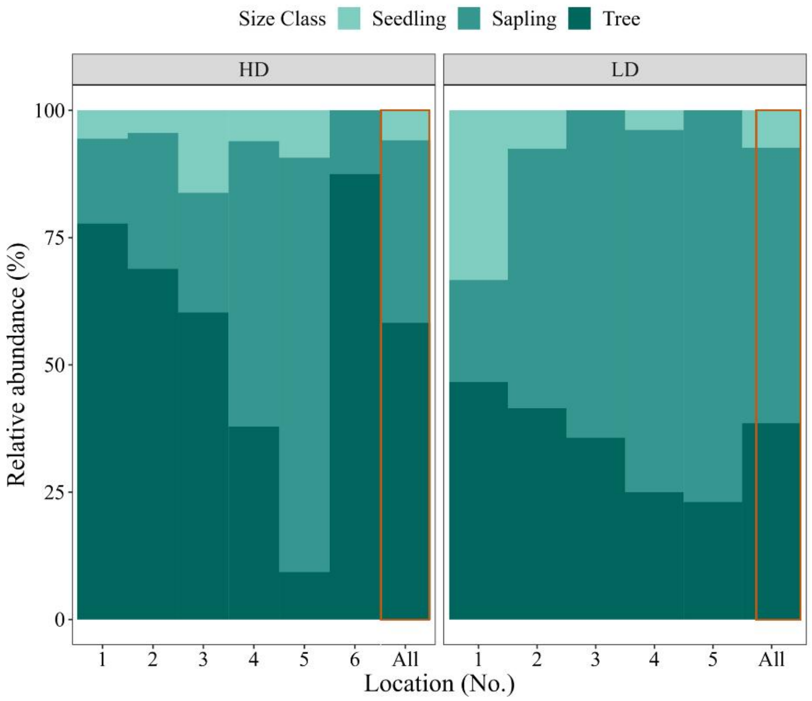

Figure S4 Comparision of regeneration status of *Rhizophora stylosa* population in heavily disturbed area (HD) and lightly disturbed area (LD) from seaward to landward (Numerically increasing, ALL represents the whole population)

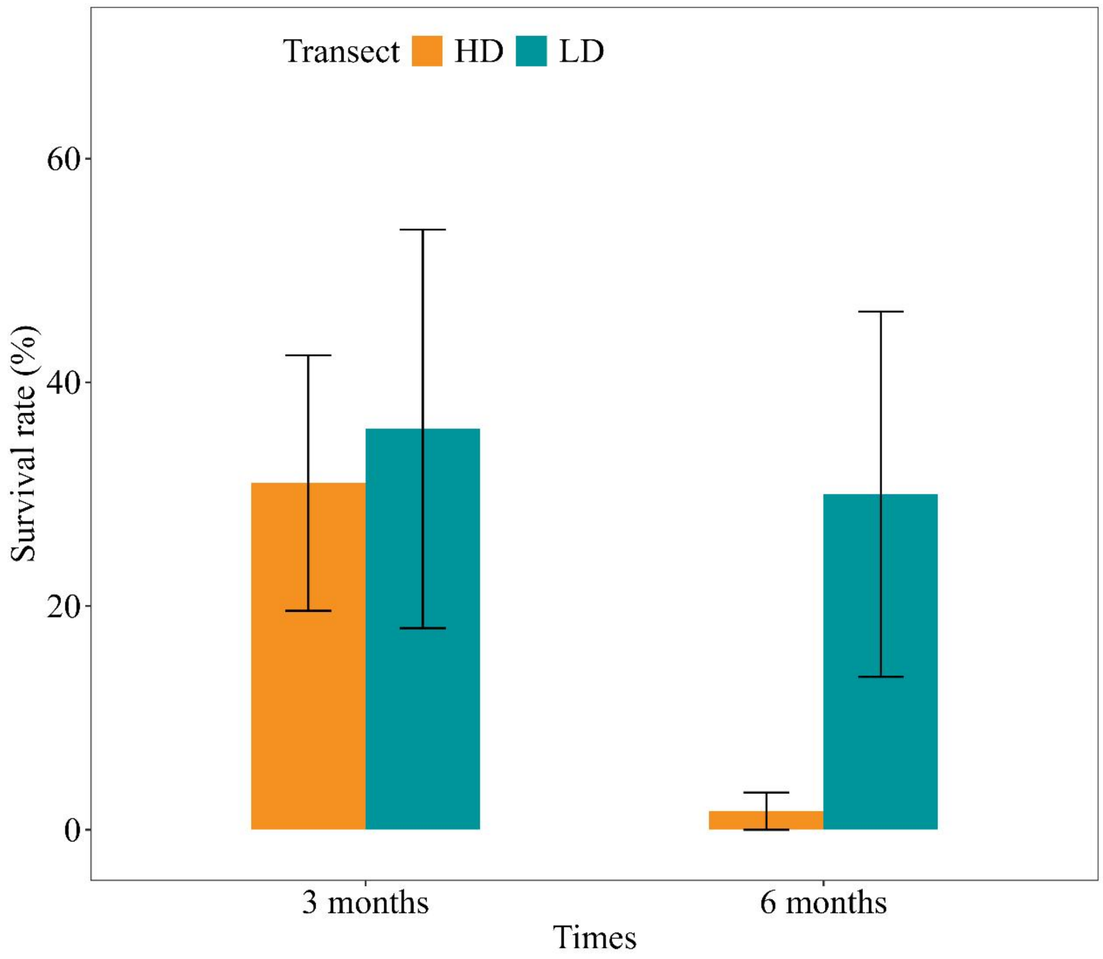

Figure S5 Comparison of survival rates of *Rhizophora stylosa* seedlings in heavily disturbed area (HD) and lightly disturbed area (LD)

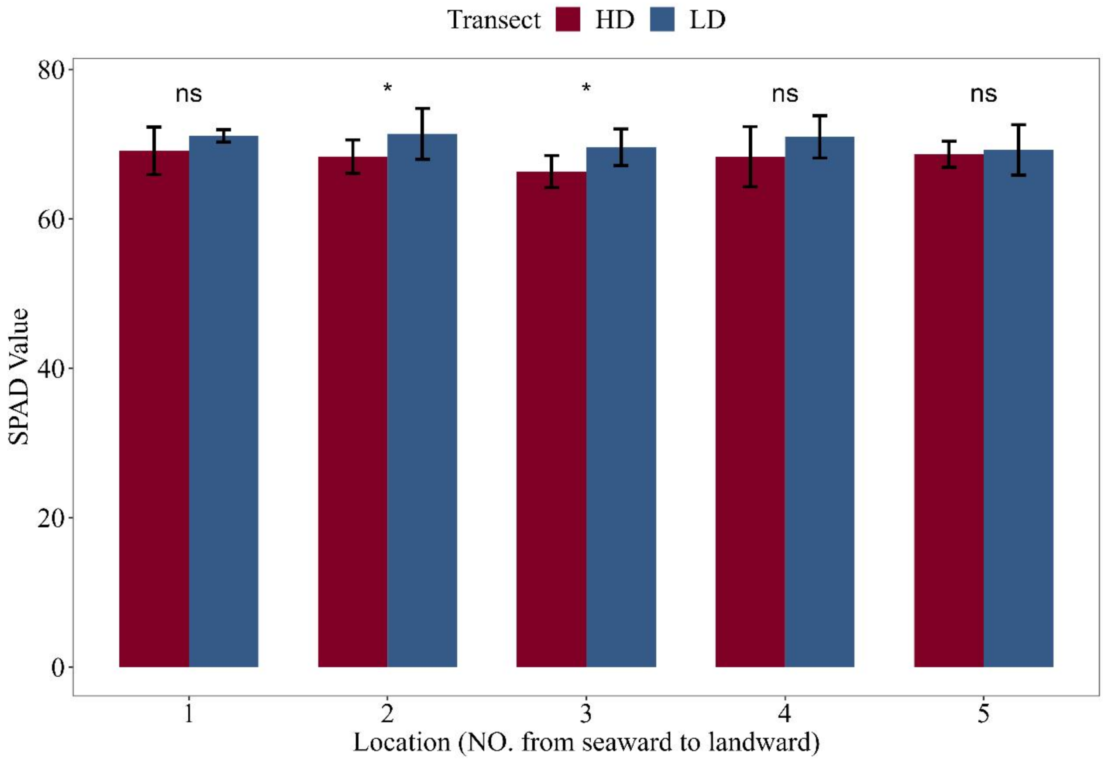

Figure S6 Differences in SPAD value of *Rhizophora stylosa* in heavily disturbed area (HD) and lightly disturbed area (LD) (Numerically increasing indicates seaward to landward)
